# The Finding of New In Vivo Metabolite Triptorelin (5-10) in Human Urine Using Liquid Chromatography Coupled with Ion Trap/Time-of-Flight Mass Spectrometry with Dimethyl Sulfoxide Additives in the Mobile Phase

**DOI:** 10.3390/molecules28124572

**Published:** 2023-06-06

**Authors:** Navaporn Saardpun, Ruamsiri Songsaeng, Pansakorn Tanratana, Thanit Kusamran, Darawan Pinthong

**Affiliations:** 1 Department of Pharmacology, Faculty of Science, Mahidol University, Bangkok 10400, Thailand; navaporn.saa@mahidol.ac.th (N.S.); pansakorn.tan@mahidol.ac.th (P.T.); 2 Analytical Science and National Doping Test Institute (ASNDTI), Mahidol University, Bangkok 10400, Thailand; ruamsiri.son@mahidol.ac.th (R.S.); thanit.kus@mahidol.ac.th (T.K.)

**Keywords:** GnRH analogs, LC/MS-IT-TOF, leuprorelin detection, triptorelin detection, triptorelin metabolite, doping

## Abstract

Triptorelin and leuprorelin are synthetic gonadotrophin-releasing hormones (GnRH) that are on the World Anti-Doping Agency (WADA) list of prohibited substances. To investigate the possible in vivo metabolites of triptorelin and leuprorelin in humans compared to previously reported in vitro metabolites, excreted urine from five patients treated with either triptorelin or leuprorelin was analyzed by liquid chromatography coupled with ion trap/time-of-flight mass spectrometry (LC/MS-IT-TOF). The addition of dimethyl sulfoxide (DMSO) to the mobile phase was found to enhance the detection sensitivity of certain GnRH analogs. The method was validated, and the limit of detection (LOD) was found at 0.02−0.08 ng/mL. Using this method, a novel new metabolite of triptorelin was discovered in the urine of all subjects up to 1 month after triptorelin administration, but it was not observed in the urine of subjects before drug administration. The limit of detection was estimated to be 0.05 ng/mL. The structure of the metabolite, triptorelin (5-10), is proposed from bottom-up mass spectrometry analysis. The discovery of in vivo triptorelin (5-10) can possibly be used as supporting evidence of triptorelin misuse in athletes.

## 1. Introduction

**Figure 1:**
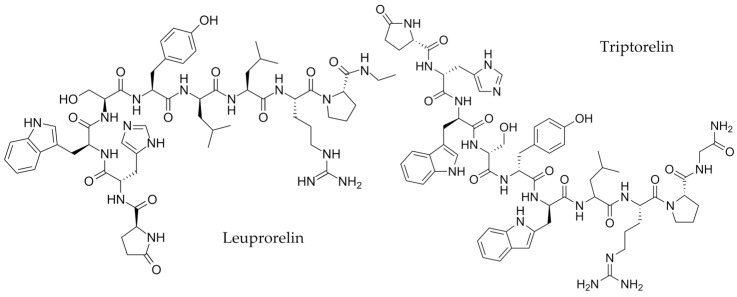
The chemical structures of leuprorelin and triptorelin.

GnRH, a 10-amino-acid peptide, is synthesized and produced in the hypothalamus [1,2,3,4]. Endogenous GnRH has a short half-life (2 min) and is readily digested by peptidases [3]. Thus, analogs such as leuprolide and triptorelin were developed to prolong their action [5]. Clinically, leuprorelin and triptorelin (Figure 1) have been mainly used for the treatment of advanced prostate cancer by androgen deprivation therapy [6,7,8]. However, the administration of GnRH analogs initially increases the testosterone level [6,9,10]. This effect has the potential to be abused by athletes in order to enhance their performance. Consequently, WADA has included GnRH analogs in its list of prohibited substances since 1st January 2016 [11].

In doping control, the determination of small peptides, including GnRH analogs, in the urine of athletes has been reported [12,13,14,15,16,17,18]. All methods employ liquid chromatography with mass spectrometric detection [12,13,14,15,16,17,18]. Sample preparation can be carried out by using solid-phase extraction (SPE) [19] or only dilution prior to liquid chromatography (LC) (the dilute-and-shoot strategy) [17]. The latter method requires a high-resolution mass spectrometer to achieve selectivity [16,17]. The detection of small peptides in doping control samples using LC/MS-IT-TOF) has been described by our group [18].

All metabolites of GnRH analogs are defined by WADA as prohibited substances, and their detection also constitutes a doping offence [11]. However, an in vivo investigation of the metabolites of triptorelin and leuprorelin has not been extensively carried out. It has been previously reported that the addition of DMSO to the mobile phase increased the sensitivity of the detection of some small peptides using electrospray ionization (ESI) [20,21,22,23,24,25]. Therefore, DMSO was used in this study for the detection of triptorelin and leuprorelin metabolites. The method for the analysis of small peptides using the LC/MS-IT-TOF instrument was validated. In this investigation, excreted urine from patients undergoing prostate cancer therapy with triptorelin or leuprorelin for the first time was screened for possible new in vivo metabolites. In this study, we hypothesized that the performance of LC/MS-IT-TOF with the DMSO additive can be achieved by the detection of the possible in vivo metabolites of these drugs in humans compared to previously reported in vitro metabolites.

## 2. Results

### 2.1. Validation Results

The method was validated in 10 different urine sources. The summary of validation results is described in Table 1. The LOD at a 95% detection rate is 0.02–0.08 ng/mL. The recovery of the validated method ranged between 58 and 86%. The matrix interference for leuprorelin, leuprorelin (5-9), and triptorelin was observed at 92%, 23%, and 16%, respectively.

### 2.2. Effect of DMSO Additive in the Mobile Phase

**Figure 2:**
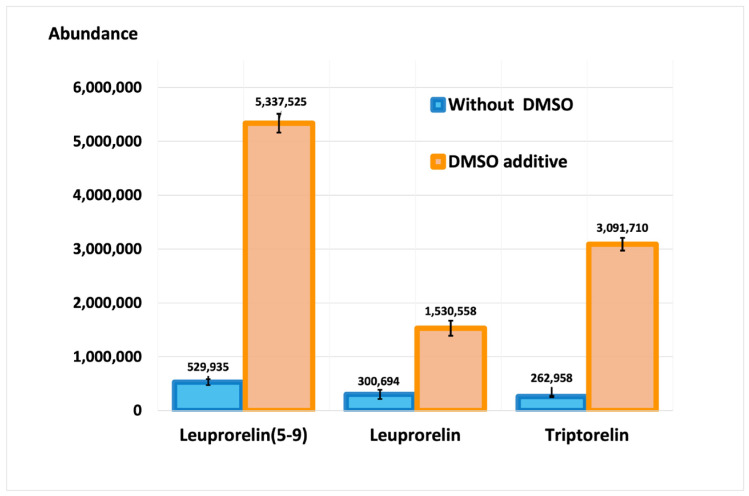
Bar graphs of the mean abundance of leuprorelin, leuprorelin (5-9), and triptorelin from the triplicate injections of a spiked urine sample at 2 ng/mL for each compound; comparison without DMSO (blue bars) and with DMSO (orange bars) additions in the mobile phase. The mean abundance was labelled on each bar with an error bar of standard deviations. The result showed an increase in signal intensity by 4 to 14 times when DMSO was added (DMSO = dimethyl sulfoxide; ng/mL = nanogram per milliliter).

The effect of DMSO was assessed by comparing the signal intensities of product ions when 1% *v*/*v* DMSO was added to mobile phase A. Figure 2 showed that the signal intensities of the three GnRH compounds increased by 4- to 14-fold.

**Figure 3:**
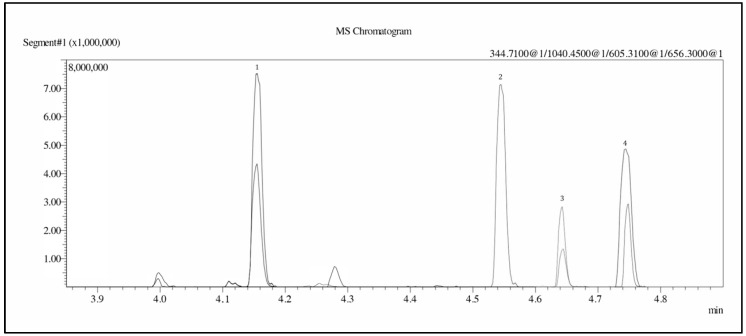
Chromatograms of the product ion transitions of leuprorelin (5-9) (peak no. 1, mass 344.71 → 249.14, 277.16), internal standard (peak no. 2, mass 1040.45 → 713.23), leuprorelin (peak no. 3, mass 605.31 → 299.21, 412.30), and triptorelin (peak no. 4, mass 656.30 → 328.20, 627.37). The precursor ions of each compound are shown in the upper–right corner. The results show the absence of an interference peak at the retention time of analytes and ISTD (ISTD = internal standard).

The method’s parameters were applied as described in Method Validation Parameters (Section 4.4). The result showed an increase in signal intensity by approximately 4, 10, and 14 times for leuprorelin, leuprorelin (5-9), and triptorelin, respectively (Figure 2). Figure 3 shows the chromatograms of the ion transitions of three standard compounds (2 ng/mL each) and the internal standard. The chromatogram showed no interference peaks at the retention times (RTs) of the analytes and internal standard.

### 2.3. Analysis of Urine from Five Prostate Cancer Patients

**Figure 4:**
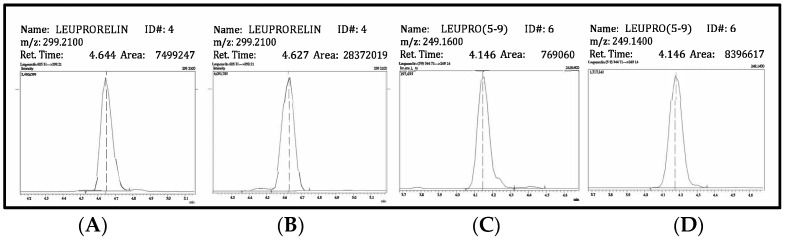
Chromatograms of leuprorelin and leuprorelin (5-9) from the urine of a prostate cancer patient collected 3 h after drug administration. (**A**,**B**) show the chromatograms of leuprorelin without and with DMSO in the mobile phase. (**C**,**D**) show the chromatograms of leuprorelin (5-9). The peak area is shown in the upper–right corner of each window. The results show 4-fold and 10-fold increases in the intensities of leuprorelin and leuprorelin (5-9) (DMSO = dimethyl sulfoxide), respectively.

Leuprorelin and its metabolite, leuprorelin (5-9), were detected in all urine samples at 3 and 6 h after leuprorelin administration. Figure 4 depicts chromatograms with and without DMSO in the mobile phase. Leuprorelin and leuprorelin (5-9) were obtained from a urine sample collected from one patient 3 h after taking the drug. The intensities of leuprorelin and leuprorelin (5-9) increased 4-fold and 10-fold, respectively, which are consistent with validation results (Figure 2). However, after 1 month, leuprorelin (5-9) was not found in the urine of all patients (Table 2).

Triptorelin and the new in vivo metabolite, triptorelin (5-10), were detected in all urine samples at 3 and 6 h after triptorelin administration (Table 3). Moreover, the new metabolite was detected in urine samples after 1 month of triptorelin administration in three out of five patients.

**Figure 5:**
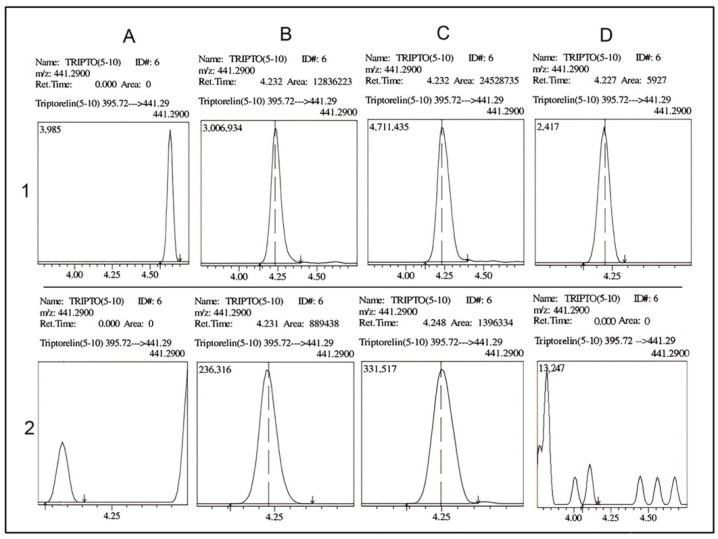
Chromatograms of triptorelin (5-10) in the urine of a prostate cancer patient. The chromatograms in columns (**A**–**C**) are from samples collected at 0, 3, and 6 h, respectively. Column (**D**) comprises the sample collected one month after administration. The first and second rows show chromatograms for the mobile phase with DMSO (row no. 1) and without DMSO (row no. 2). The results show the detection of triptorelin (5-10) in all urine and up to 1 month after drug administration in the presence of DMSO (DMSO = dimethyl sulfoxide).

In the presence of DMSO, the chromatograms indicated the presence of in vivo triptorelin (5-10) in all urine samples collected 3 h, 6 h, and 1 month after drug administration (Figure 5(1B–1D)). Without DMSO, triptorelin (5-10) was detected in lower concentrations in collected urine (Figure 5(2B,2C)), and the detection after one month of drug administration could not be achieved (Figure 5(2D)). As shown in Figure 5, the addition of DMSO to mobile phase A increased the intensities of both triptorelin and triptorelin (5-10). The 14-fold increase in triptorelin intensity is consistent with validation results (as in Figure 2).

### 2.4. Detection and Identification of In Vivo Triptorelin Metabolite

**Figure 6:**
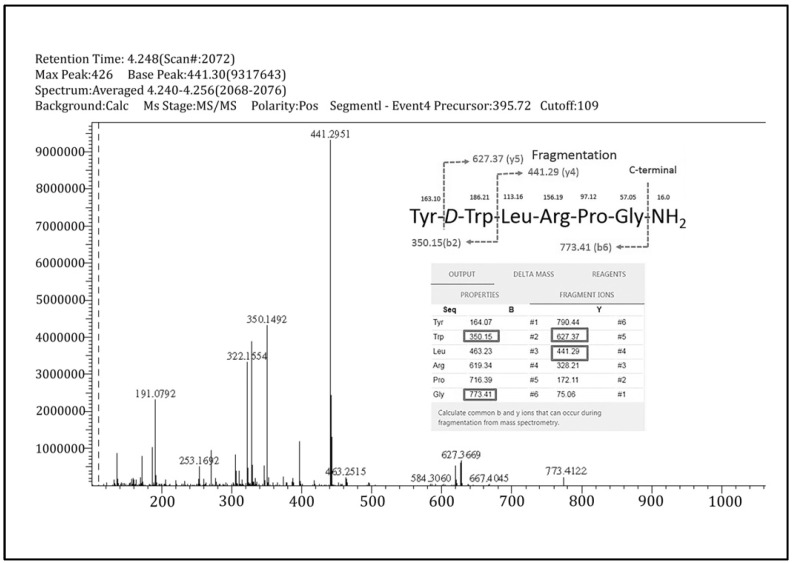
The mass spectrum of triptorelin (5-10) for precursor ion *m*/*z* 395.72 ([M + 2H]^2+^). The major fragments were *m*/*z* 441.29, 350.15, 627.37, and 773.41, which were identified as y_4_, b_2_, y_5_, and b_6_ ions. The amino acid sequence and proposed fragmentation scheme were compared with the Peptide Calculator’s result. The fragmentation of in vivo triptorelin (5-10) was proposed based on bottom-up mass spectrometry analysis.

The in vivo detection of the triptorelin (5-10) metabolite has not been reported previously. In the initial study, all urine samples collected from five patients were extracted and analyzed using the full-scan mode of LC/MS-IT-TOF. This was carried out to check if there were any potential new peaks that were not present in the 10 normal urine samples used in the validation study of the 3 GnRH analogs. It was clearly shown that there was a peak at RT 4.240–4.256 min in all collected urine samples after 3 and 6 h of drug administration. Figure 6 depicts the mass spectrum of the peak. A preliminary finding of the new in vivo metabolite of triptorelin, triptorelin (5-10), was detected, and its structure was proposed based on the bottom-up mass spectrometry analysis [26,27] accordingly.

According to mass spectrometric data of the new finding of in vivo triptorelin (5-10) (Figure 6), base ion *m*/*z* 395.72 is shown later to be a doubly charged molecule [M + 2H]^2+^ of triptorelin (5-10) with a pseudomolecular ion [M + H]^1+^ of *m*/*z* 790.43. The product ion mass spectrum of precursor ion *m*/*z* 395.72 is observed. The fragmented ions are *m*/*z* 441.29 (relative abundance = 100%), 350.15 (relative abundance = 50%), and 627.37 (relative abundance = 10%); and 773.41 (relative abundance = 5%).

## 3. Discussion

DMSO has previously been used in proteomics to enhance electron ionization [20,21,22,23,24,25]. One study showed that 1–5% DMSO in the mobile phase resulted in an increase in the sensitivity of GnRH detection by approximately 2- to 15-fold [15]. The use of DMSO as an additive in the electrospray ionization of small peptides using an Orbitrap instrument and dilute-and-shoot method resulted in an increase in ion abundance from 3- to 5-fold [17]. Compared to this study, the DMSO additive using LC/MS-IT-TOF and solid phase extraction resulted in an increase in ion abundance from 4- to 14-fold. The effect of DMSO depends on several factors, including sample preparation and the mass analyzer model [22]. In the proteomic experiment, a comparison of different ESI mass analyzer models, including three models of Orbitrap, TOF, Quadrupole-TOF, and Ion Mobility-TOF, revealed varying degrees of efficiency with the DMSO additive [22]. Due to the accurate mass measurement and multiple stages of ionization mass analysis (MS/MS mode), LC/MS-IT-TOF was previously suitable for performing qualitative analysis and was commonly used for unknown identifications in several fields, including herbal medicine, pesticides, protein, and peptides, in biological and biomedical analyses [26,27,28]. From this study, the combination of LC/MS-IT-TOF with the DMSO additive increased the advantage of this instrument, and the instrument went from performing qualitative to quantitative analyses with respect to low concentrations of new metabolites, which fitted the purpose of our study.

The validation results of the method developed in this study with the DMSO additive exhibited increased sensitivity and performance for detecting in vivo metabolites using LC/MS-IT-TOF [18]. No interference peak was observed from the analysis of the reagent blank and the mixture of 3 analytes in 10 different blank urine; therefore, the selectivity of this method was accepted. The shift in retention time was observed at ± 0.05 at most, and the relative retention time (RRT) was stable. There was no indication of the peak deformation caused by the urine matrix. As shown in Table 1, the instrument’s precision is less than 15%. Despite the relatively large injection volume (30 μL), carryover was not observed in blank urine injected after 16 ng/mL of spiked urine samples was injected from 10 different sample sources. The intensity of the triptorelin (5-10) metabolite increased 14–17 fold, which is substantially greater than the intensity of leuprorelin (5-9). This significant increase in sensitivity enabled the possibility of the detection of triptorelin (5-10) after one month of drug administration (compared to Figure 5(1D,2D)). Therefore, the application of LC/MS-IT-TOF and the DMSO additive was efficient for detecting the low-concentration metabolite, and this may not have been achieved when using standalone LC/MS-IT-TOF. According to the findings of this study, DMSO-enhancing ionization in LC/MS-IT-TOF achieved the sensitivity required in doping control. This application allowed us to use this kind of instrument to access other drug metabolites.

Triptorelin (5-10) had previously been found in an in vitro investigation using human kidney microsome cell cultures [13]. The amino acid sequence of this metabolite discovered in vivo was likely identical to that found in vitro. In terms of mass fragmentation, based on bottom-up mass spectrometric analysis, which is commonly used for drug identification [29,30], masses of *m*/*z* 441.29, 350.15, 627.37, and 773.41 were identified as y_4_, b_2_, y_5_, and b_6_ ions. This is related to the result from Peptide Calculator software (version 2.5 Beta), as illustrated in Figure 6. This finding is the preliminary result of this novel metabolite and the projected amino acid sequence. Additional experiments to confirm the structure of this metabolite can be achieved by matching the chromatographic and mass spectrometric data of synthetically made triptorelin (5-10).

The peak that was identified as the triptorelin metabolite comprised elutes at the expected RT in all urine samples from all patients 3 h after drug administration (refer to Table 3), and the elutes exhibited high amounts, with the highest estimated concentration at 225.7 ng/mL and the lowest estimated concentration at 12.8 ng/mL. However, none of these finding peaks were observed in the urine samples prior to drug administration. Therefore, presence of triptorelin (5-10) confirmed the existence of a metabolite from triptorelin ad-ministration.

Previous literature described peptide metabolism via peptidases found in multiple organs, such as the lung, blood, kidney, skin, and epithelial cells [31,32], but the metabolism of triptorelin remains unknown. The finding of this in vivo metabolite may be due to a particular cleavage from triptorelin to triptorelin (5-10), also involving a cleavage from leuprorelin to leuprorelin (5-9) at the same position (serine is linked to tyrosine). The metabolism study model using peptidases in several cell cultures is intriguing and can be used to learn more about GnRH metabolism.

Although triptorelin (5-10) was not found in two out of five patients after 1 month of drug administration, this could be due to the concentration below the LOD. The detection period of triptorelin (5-10) is, however, longer than the parent compound itself. It is possible to use this metabolite as a marker and/or supportive evidence for the misuse of triptorelin by athletes. Additionally, the monitoring of this metabolite may also be useful in clinical treatment. The link between triptorelin (5-10) concentrations, luteinizing hormone (LH) concentrations, patient symptom improvement, and the adverse effect of these medicines remains to be investigated.

## 4. Materials and Methods

### 4.1. Chemicals and Materials

Leuprorelin and triptorelin were purchased from BACHEM (Bubendorf, Switzerland). The leuprorelin (5-9) metabolite was supplied by NMI (New South Wales, Australia). Deamino-Cys^1^-Val^4^-D-Arg^8^-vasopressin was obtained from Sigma-Aldrich (Burlington, MA, USA) and used as the internal standard (ISTD). Acetic acid glacial (CH_3_COOH) and sodium dihydrogen phosphate (NaH_2_PO_4_·H_2_O) were purchased from Carlo Erba (Peypin, France). Formic acid (HCOOH) and methanol (CH_3_OH) of HPLC (high-performance liquid chromatography) grade were purchased from Fisher Chemical (Pittsburgh, United States). Acetonitrile was purchased from RCILabscan (South Australia, Australia). Disodium hydrogen phosphate (Na_2_HPO_4_) was purchased from QRec (Auckland, New Zealand). DMSO was purchased from RCI Labscan (Taipei City, Taiwan). Mixed-mode weak cation exchange cartridges Oasis^®^ WCX (60 mg, 3 mL) and SPE were purchased from Waters (Milford, MA, USA). Protein LoBind^®^ centrifuge tubes (0.5, 1.5, 2.0, and 5.0 mL) were purchased from Eppendorf (Melbourne, Australia). Polypropylene vials (250 mL and 1.0 mL) and Teflon-lined caps were purchased from Agilent (Santa Clara, CA, USA). The vacuum centrifuge concentrator was from Eppendorf (Taufkirchen, Germany). Bench-top refrigerated centrifuge model 5930 was supplied by Kubota (Tokyo, Japan). Ultra-pure water purification system model Superseries PW was supplied by Heal Force (Shanghai, China).

### 4.2. LC/MS-IT-TOF Instrument

Sample analysis was carried out by using the LCMS-IT-TOF Prominence system (Shimadzu, Japan) and LC model UPLC 8040 [18]. The analytical column was a Poroshell 120 EC-C18 (2.1 mm × 150 mm, 2.7 μm particle) coupled with a guard column (SecurityGuard^TM^ C18, 3.0 mm). The column compartment temperature was set at 30 °C. The autosampler unit temperature was set at 10 °C. Dry gas was set at 47 kPa, and nebulizer gas was set at 1.5 L/min. The curved desolvation line (CDL) and heating block temperature were set at 250 °C. The spray voltage was set at 4 kV.

### 4.3. Standard Solutions

A standard mixture of leuprorelin, leuprorelin (5-9), and triptorelin (1 mg/mL each) was prepared in 0.1% acetic acid in a polypropylene vial. All standard solutions were aliquoted and stored at −70 °C for individual use. A working solution of 10 μg/mL deamino-cys^1^-val^4^-D-arg^8^-vasopressin prepared in acetic acid was used as the internal standard (ISTD).

### 4.4. Method Validation Parameters

The method was validated in accordance with ISO/IEC17025 requirements [33] for the qualitative analysis of mass spectrometry in terms of LOD, selectivity, recovery, matrix effect, instrument precision, and carryover. The validation of leuprorelin, leuprorelin (5-9), and triptorelin was performed using 10 different sources of urine samples.

LOD was established by identifying the minimum concentration of each detected compound. The spiked urine was prepared at 0, 0.01, 0.1, 0.2, 1.0, and 2.0 ng/mL. A signal-to-noise ratio (S/N) of 3 or higher was used as the acceptance criteria for detection. Selectivity was evaluated by the separation of analyte peaks from neighboring peaks at the expected retention time in samples. Recovery was assessed at 2 ng/mL from three replicated samples. The percentage of recovery was calculated by comparing the peak area ratio of each compound to ISTD in the pre-sample (spike urine extraction) with a post-sample (spiked standard in extracted blank urine at the same amount).

The matrix effect and instrument precision were assessed at 2.0 ng/mL in three replicated samples. The percentage of the matrix effect was determined by comparing the peak area ratio of each compound to ISTD in the direct standard with a post-sample at equal concentrations. Precision was determined by calculating the percent coefficient of variation (%CV) of the product ion intensity of each compound from 10 consecutive injections. Per the FDA’s “Bioanalytical Method Validation Guidance for Industry” [34], the acceptance criteria for determining the efficiency of the instrument is when %CV is not greater than 15%. Carryover was assessed by injecting blank urine immediately after 16.0 ng/mL (8 times of 2 ng/mL, the minimum required performance limit) of the analytes spiked in each urine source was injected. The absence of detected compounds in all blank urine samples is the acceptance criterion.

### 4.5. Collection and Storage of Urine Samples

Urine samples were collected at the Division of Urology, Department of Surgery, Faculty of Medicine, Ramathibodi Hospital, Bangkok, Thailand. All subjects gave their informed consent for inclusion before they participated in this study. The study was conducted in accordance with the Helsinki Declaration, and the protocol was approved by the Ethics Committee review board of Ramathibodi Hospital, Bangkok, Thailand (protocol No. 12-61-53). Urine samples were collected at 0.0, 3.0, and 6.0 h from 5 patients who had received either 11.25 mg of Enantone^®^ L.P (leuprorelin acetate) or Diphereline^®^ P.R. (triptorelin pamoate) intramuscularly for the first time. A further urine sample was collected one month later.

Urine samples were kept at −70 °C for long-term storage as it had been found that there was a gradual decrease in peptide metabolites after six months of storage at −20 °C [35]. Urine analyses were performed as soon as possible after the urine samples were thawed.

### 4.6. Urine Sample Preparation

In this study, 200 mL of sodium phosphate buffer (pH 6.8; 0.8 M) was added to a 3.0 mL aliquot of urine. The batch of samples was centrifuged at 5000 rpm for 5 min at 5 °C. The supernatant from each tube was transferred to a new set of protein LoBind^®^ tubes, Eppendorf (Melbourne, Australia) and 20 μL of ISTD (see Section 4.3) was added to each sample [17]. SPE cartridges (see Section 4.1) were activated with 2 mL of methanol followed by 2 mL of ultra-pure water. The supernatants were slowly loaded onto the SPE cartridges (flow rate is approximately 1.0 mL/min) and washed with 1 mL of water, followed by 1 mL of freshly prepared 10% *v*/*v* aqueous methanol; then, all washing solutions were removed and dried using a vacuum pump. Elution was performed using freshly prepared 10% *v*/*v* formic acid in methanol. Eluates were evaporated at 45 °C to approximately 10 μL in a vacuum centrifuge. The residues were reconstituted in 50 μL of freshly prepared 0.1% formic acid in water.

### 4.7. LC/MS-IT-TOF Conditions

The mobile phases were 0.1% formic acid in water with or without 1% DMSO (mobile phase A) and acetonitrile (mobile phase B) at a flow rate of 0.3 mL/min. The mobile phase gradient program was as follows: 0.0–2.0 min, 20 to 30% B; 2.0–4.0 min, 30 to 45% B; 4.0–5.0 min, 45 to 100% B; 5.0–6.0 min, hold at 100% B; 6.0–7.0 min, 100% to 20% B; and 7.0–9.0 min hold at 20% B (column equilibration). The injection volume was 30 μL. The total runtime is 9 min.

Mass spectrometric analysis was employed both in the full-scan mode and MS-MS mode. Data analysis was performed using 2 product ion transitions for each compound. LC/MS-IT-TOF mass spectrometric variables, including the *m*/*z* of product ions, ion accumulation time, and collision energy (CE), were optimized for maximum abundance for each ion transition. Ionization was performed using ESI in the positive ion mode at 3500 V. The ion accumulation time was 10 milliseconds, and the isolation width was 1.5 Dalton.

The chromatographic and mass spectrometric data for the detection of all 4 peptides and the internal standard are listed in Table 4.

### 4.8. Statistic Analysis

The mean and standard deviation of the concentration of leuprorelin, leuprorelin (5-9), triptorelin, and triptorelin (5-10) from 5 patients were used for statistical analysis. The plot of mean ± SEM (*n* = 5) was provided for Table 2 and Table 3 (SEM = standard error of mean).

## 5. Conclusions

The application of LC/MS-IT-TOF with the DMSO additive was efficient for the detection of a low-concentration metabolite, and this may not have been achieved when using standalone LC/MS-IT-TOF. In this study, triptorelin (5-10), a new in vivo metabolite of triptorelin, was detected in the urine of prostate cancer patients using LC/MS-IT-TOF. The addition of 1% DMSO to the mobile phase improved the sensitivity of the detection of these peptides, making it possible to detect low concentrations of the in vivo triptorelin (5-10) metabolite for up to one month after drug administration, which was not observed before drug administration. For doping analysis, the sensitivity and the longer detectable period provide an advantage for drug abuse detection in sports. Thus, triptorelin (5-10) could potentially be used as a possible marker and supporting evidence of the misuse of triptorelin in sports. This application allowed us to use this instrument to access other peptide metabolites.

## Figures and Tables

**Table 1:** Summary of the validation results for the detection of leuprorelin, leuprorelin (5-9), and triptorelin using LC/MS-IT-TOF. LOD was compared in the absence (LOD ^1^) and presence (LOD ^2^) of DMSO. The LOD was calculated at a 95% detection rate from the sigmoid response curve. The result showed the LOD is lower in the presence of DMSO than it is in the absence of DMSO. (%CV = percent coefficient of variation; LOD = limit of detection; DMSO = dimethyl sulfoxide; ng/mL = nanogram per milliliter; LC/MS-IT-TOF = liquid chromatography coupled with ion trap time-of-flight mass spectrometer).

Compounds	Without DMSO	DMSO Additive			
	LOD ^1^ (ng/mL)	LOD ^2^ (ng/mL)	Recovery (%)	Matrix Effect (%)	Instrument Precision (%CV)
Leuprorelin	0.35	0.08	85	92	11
Leuprorelin (5-9)	0.20	0.02	64	23	3
Triptorelin	0.60	0.05	58	16	12

**Table 2:** The detection level (ng/mL unit) of leuprorelin and leuprorelin (5-9) in urine samples collected from 5 patients 3 h, 6 h, and 1 month after drug administration. The results showed that leuprorelin and leuprorelin (5-9) could be detected in all urine samples collected at 3 and 6 h but not at 1 month after drug administration (ND = not detectable; ng/mL = nanogram per milliliter; SEM = standard error of mean).

Patient	Leuprorelin			Leuprorelin (5-9)		
No.	3 h	6 h	1 Month	3 h	6 h	1 Month
1	29.7	86.9	ND	68.0	73.1	ND
2	96.3	29.0	ND	106.9	18.7	ND
3	86.5	66.2	ND	162.9	100.4	ND
4	51.2	131.6	ND	16.7	23.8	ND
5	61.5	217.1	ND	19.3	178.7	ND
mean	65.0	106.2	ND	74.8	78.9	ND
n	5	5	5	5	5	5
SEM	12.0	32.2	-	27.6	29.2	-

**Table 3:** The detection level (ng/mL unit) of triptorelin and triptorelin (5-10) in urine samples collected from five patients 3 h, 6 h, and 1 month after drug administration. The results show that triptorelin and triptorelin (5-10) can be detected in all urine samples collected at 3 and 6 h, as well as up to 1 month in 3 out of 5 patients (ND = not detectable; ng/mL = nanogram per milliliter; SEM = standard error of mean).

Patient	Triptorelin			Triptorelin (5-10)		
No.	3 h	6 h	1 Month	3 h	6 h	1 Month
1	33.4	16.9	ND	84.3	24.7	1.2
2	22.1	24.6	ND	25.3	43.5	0.3
3	32.1	54.4	ND	12.8	3.2	ND
4	81.3	104.9	ND	225.7	250.6	ND
5	151.8	112.2	ND	179.1	202.0	0.6
mean	64.1	62.6	ND	105.5	78.9	0.7
n	5	5	5	5	5	3
SEM	24.2	19.8	-	42.0	29.2	0.2

**Table 4:** Chromatographic and mass spectrometric data for the detection of target compounds and the internal standard using LC/MS-IT-TOF (g/mol = gram per mol; min = minute; *m*/*z* = mass per charge; eV = electron volt; ISTD = internal standard).

Compound	Chemical Formula (g/mol)	Retention Time (min)	Monoisotopic Mass (*m*/*z*)	Precursor Ion (*m*/*z*) ^(1)^	Product Ion (*m*/*z*)	Collision Energy (eV)
Leuprorelin	C_59_H_84_N_16_O_12_	4.65	1208.64	605.31^2+^	299.21^1+^, 412.30^1+^	27
Leuprorelin (5-9)	C_34_H_57_N_9_O_6_	4.16	687.45	344.71^2+^	249.14^1+^, 277.16^1+^	30
Triptorelin Triptorelin (5-10) ^(2)^ ISTD	C_64_H_82_N_18_O_13_ C_39_H_55_N_11_O_7_ C_46_H_65_N_13_O_11_S_2_	4.76 4.24 4.55	1310.63 790.43 1040.22	656.30^2+^ 395.72^2+^ 1040.45^1+^	328.20^1+^, 627.37^1+^ 441.29^1+^, 350.15^1+^ 713.23^1+^	50 25 40

^(1)^ Charged state of the ion. ^(2)^ Mass spectrometric data from excretion urine (see Section 2.4).

## Data Availability

Not applicable.
